# Step by step Indiana pouch construction in a previously irradiated patient with a cervical cancer relapse

**DOI:** 10.1016/j.ijscr.2019.11.068

**Published:** 2019-12-09

**Authors:** Antoni Llueca, Yasmine Maazouzi, Paula Ponce, Anna Serra, Carmen Garau, Miguel Rodrigo

**Affiliations:** a Multidisciplinary Unit for Abdominal Pelvic Oncology Surgery (MUAPOS), University General Hospital of Castellon, Spain; b Dept. of Medicine, University Jaume I (UJI), Castellon de la Plana, Spain

**Keywords:** Cervical cancer, Previously irradiated pelvis, Indiana Pouch (IP), Step by step, LEER

## Abstract

**Introduction:**

Radiation therapy and radical pelvic surgery, either radical cystectomy or pelvic exenteration, is the golden standard treatment for infiltrating bladder carcinoma, as well as advanced or recurrent cervical, vulvar, vaginal and endometrial cancer. However, due to the poor radiation sensitivity of the cervix and vagina, a high-radiation dose is required, leading to early and/or late onset urogenital complications in approximately 50% of the patients.

**Case presentation:**

The following case report describes a 64-year-old native Russian woman presenting a relapse of a vaginal cuff squamous cell carcinoma, who underwent a laterally extended endopelvic resection (LEER) followed by a neobladder reconstruction based on the Indiana pouch (IP) technique. The process is described here step by step.

**Discussion:**

Indiana pouch urinary diversion was based on thorough research, the reproducibility of the technique, our urologist’s experience with the Indiana Pouch, as well the lower rate of complications published in various separate series.

**Conclusion:**

Indiana pouch is a successful continence urinary reservoir with a reproductible technique, however long-term observation is needed.

## Highlights

• Radical pelvic surgery is the golden standard surgical treatment of pelvic malignances and urinary diversion is often needed.
• Catheterizable continent urinary derivation (CCUD) is one of the best options in young patients.
• Indiana Pouch is a reproductible technique with low rate of complications.

## 1 Introduction

Radical pelvic surgery, either radical cystectomy or pelvic exenteration, is the golden standard surgical treatment for infiltrating bladder carcinoma, as well as advanced or recurrent cervical, vulvar, vaginal and endometrial cancer. Additionally, such patients usually receive radiotherapy. However, due to the poor radiation sensitivity of the cervix and vagina, a high-radiation dose is required, leading to early or late urogenital complications in approximately 50% of the patients [[1], [2], [3]]. Up to 17% of the patients with a history radiated cervical cancer eventually present a central pelvic recurrence, making them candidates for an anterior pelvic exenteration and urinary diversion [4]. The concept of urinary diversion dates back to the nineteenth century and had many uses. The first urinary diversion, a ureterosigmoidostomy, was performed by Simon in 1852 in children with bladder exstrophy. The historical breakthrough occurred in 1950, where Bricker et al. described the ileal conduit, which is still being used nowadays, following a radical pelvic surgery [5]. In 1970, Kock et al., the founder of catheterizable continent urinary derivations (CCUD), developed the Kock Pouch (ileo-anal reservoir) for patients requiring a total colectomy, presenting for the first time the concept of detubulization [6]. By 1980, Hautmann et al. adapted the previously mentioned Kock pouch, adding a urethral anastomosis, creating the orthotopic neobladder, which quickly gained popularity in the 1990’s [7]. More recently, the University of Indiana presented in 1987 its own catheterizable continent urinary derivation (CCUD), described by Rowland et al. as an alternative to the orthotopic neobladder, which is still used nowadays and will be our adopted technique for the patient described in this case report.

Our choice of urinary derivation was based on thorough research, the reproducibility of the technique, our urologist’s experience with the Indiana Pouch (IP), the lower rate of complications published in various separate series, such as Houvenaeghel et al. and Castillo et al., describing the use of the technique in previously radiated patients with advanced gynecological cancers [8,9]; and finally the advantages of a CCUD over the orthotopic neobladder such as a better nighttime and early daytime continence [10].

In the following case report, we describe the IP technique step by step, performed by a multidisciplinary team of a gynecologist, a general surgeon and a urologist, immediately following a laterally extended endopelvic resection (LEER). This work has been done according to the SCARE criteria [11].

## 2 Case presentation

The patient is a 64-year-old native Russian woman, currently living in Spain, presenting a relapse of a vaginal cuff squamous cell carcinoma. She is allergic to macrolides and chloramphenicol, she underwent appendectomy, and has an obstetrical history of two natural births. In 2011, the patient was diagnosed with an endometrial carcinoma (FIGO II) which was treated through a hysterectomy and bilateral adnexectomy, via laparotomy. Later, she received adjuvant external beam radiotherapy (a total dose of 45 Gy). On March 2015, she was diagnosed with vaginal cuff squamous cell carcinoma which was resected and was additionally treated with brachytherapy. The previously mentioned interventions were all performed in Russia. On January 2016, the patient comes to our consultation, presenting a 4 cm vaginal cuff lesion which was biopsied, resulting in a well-differentiated infiltrating squamous carcinoma. The patient’s preoperative blood work was normal, including the renal function, and the thoraco-abdomino-pelvic scan revealed implants at the base of the bladder. On February 2016, the patient is intervened and a laterally extended endopelvic resection (LEER) with a continent urinary diversion (IP) is performed.

## 3 Operative technique

**Fig. 1:**
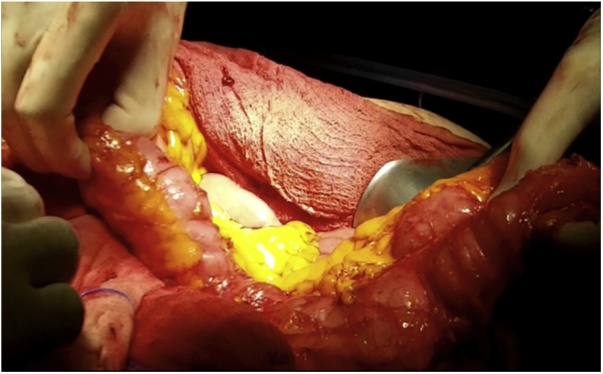
Identification of the bowel segment used for the Indiana Pouch.

**Fig. 2:**
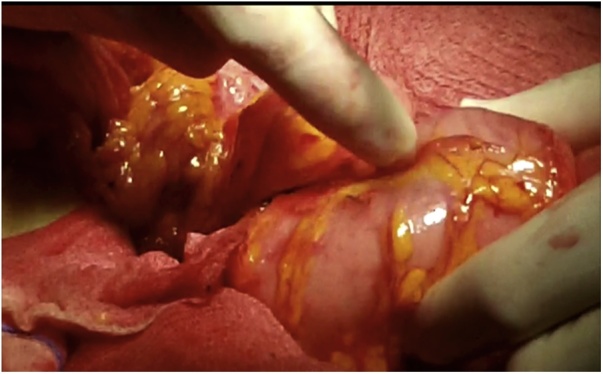
Resection of the ascending colon to the right of the middle colic artery, and the terminal ileum approximately 15–20 cm away from the ileocecal valve.

**Fig. 3:**
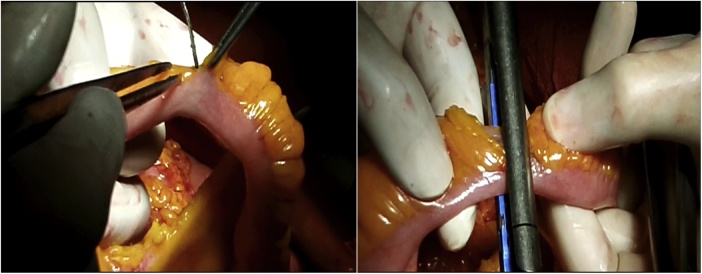
Sectioning of terminal ileum, approximately 15–20 cm away from the ileocecal valve.

**Fig. 4:**
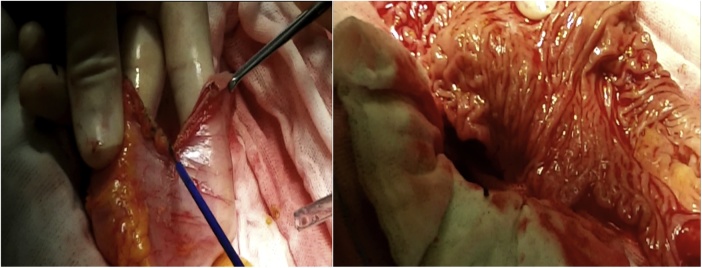
The right colon segment is detubularized along the taenia coli using an electric scalpel.

**Fig. 5:**
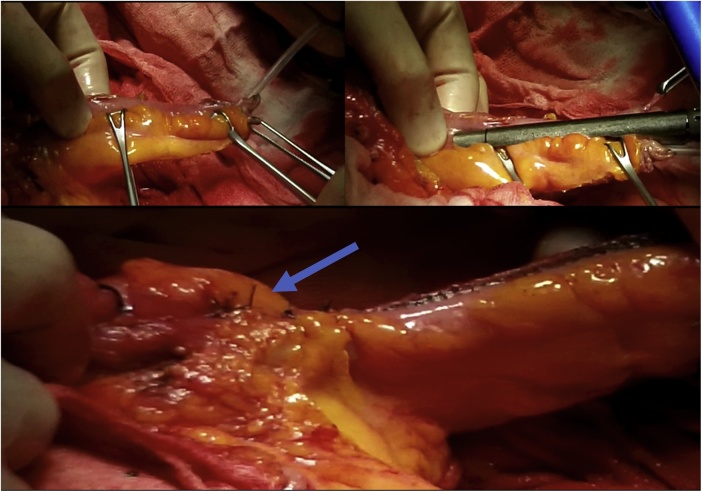
A 12 French Nelaton catheter is introduced inside the ileal segment, which is then tapered over using a GIA stapler, forming a “pseudo-appendix”. Imbricating sutures (arrow) are placed at the ileocecal valve in order to ensure the smooth catheterization of the channel.

**Fig. 6:**
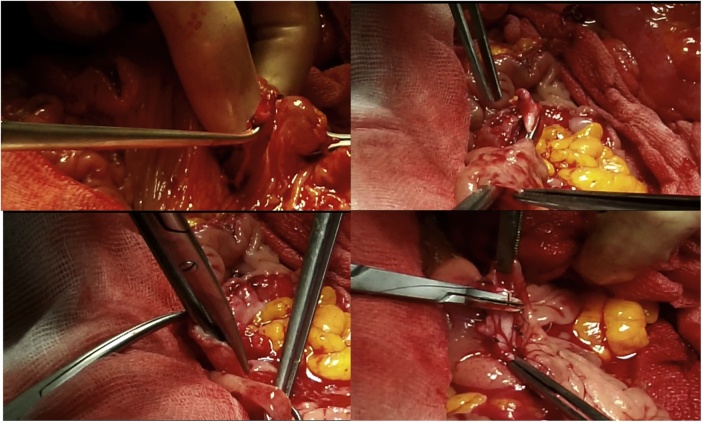
The left ureter is crossed over to the right, passing under the mesosigma, and spatulated. A direct mucosae to mucosae anastomosis is performed (ureteroenteric anastomosis) using 5-0 monofilament simple sutures. The same is done with the contralateral ureter.

**Fig. 7:**
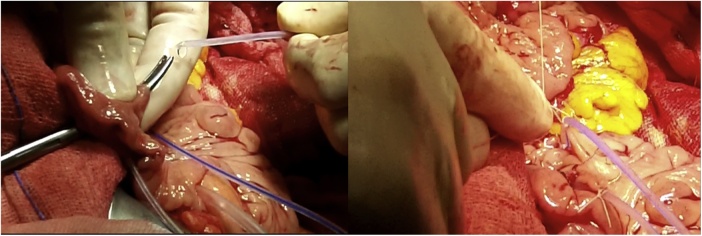
The ureteroenteric anastomosis are then stented using 8 French catheters, and led out of the pouch and through the abdominal wall (in the right flank), lateral to the medial laparotomy incision.

**Fig. 8:**
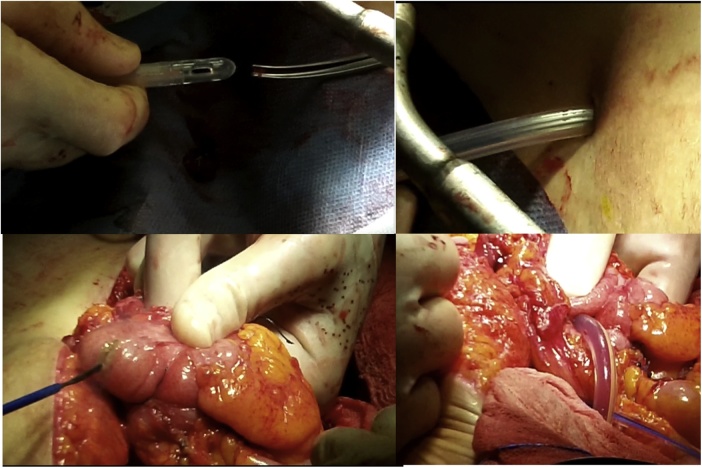
A Malecot or Pezzer 20 French catheter is inserted through the right abdominal flank, and into the pouch, securing it with a purse-string suture, and will serve as a cystostomy drain.

**Fig. 9:**
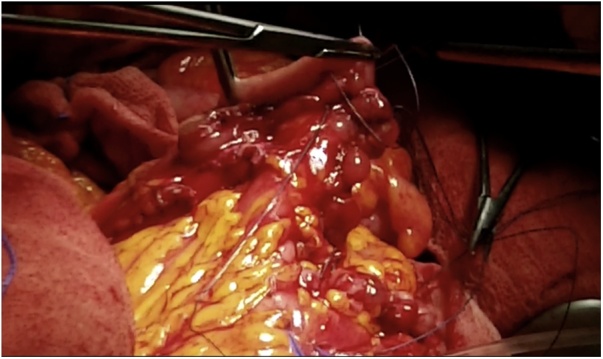
The rest of the pouch is then sutured in a spherical reservoir using the 3-0 monofilament continuous suture.

**Fig. 10:**
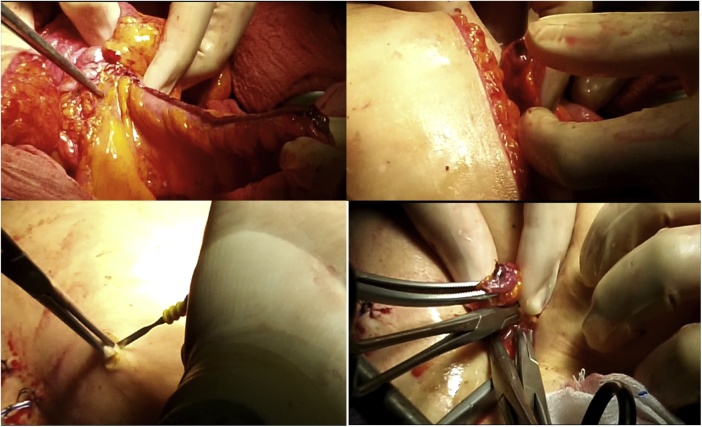
In the right pelvic region, the previously catheterized and tapered ileum is exteriorized.

**Fig. 11:**
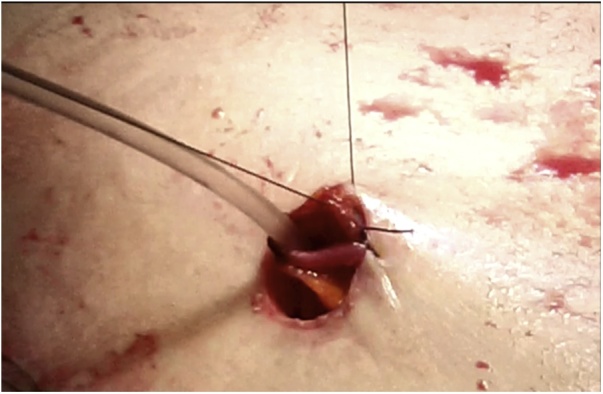
The exteriorized ileum is fixed to the skin using simple vycril 3-0 sutures.

Once the LEER is performed according to our usual technique, the IP is constructed during the second half of the surgical procedure. The first step of the procedure consists in sectioning the ascending colon, to the right of the middle colic artery, and the terminal ileum approximately 15–20 cm away from the ileocecal valve. The reconstruction of the bowel was done using a termino-lateral ileocecal anastomosis using a GIA stapler. The isolated right colon segment is then detubularized along the taenia coli using an electric scalpel and shaped into a “U” configuration, and one of the sides is sutured with a continuous, absorbable suture (3-0 monofilament). A 12 French Nelaton catheter is introduced inside the ileal segment, which is then tapered over using a GIA stapler, forming a “pseudo-appendix” (Fig. 1, Fig. 2, Fig. 3, Fig. 4, Fig. 5, Fig. 6). Imbricating sutures are placed at the ileocecal valve in order to ensure the smooth catheterization of the channel. The left ureter is crossed over to the right, passing under the mesosigma, and spatulated. Direct mucosae to mucosae anastomosis is performed (ureteroenteric anastomosis) using 5-0 monofilament simple sutures. The same is done with the contralateral ureter. Spontaneous urine ejaculation is witnessed before moving on, in order to confirm the normal, unobstructed ureteral course. The ureteroenteric anastomoses are then stented using 8 French catheters, and led out of the pouch and through the abdominal wall (in the right flank), lateral to the medial laparotomy incision (Fig. 7). A Malecot or Pezzer 20 French catheter is selected and is inserted through the right abdominal flank, and into the pouch, securing it with a purse-string suture whichwill serve as a cystostomy drain (Fig. 8) The rest of the pouch is then sutured in a spherical reservoir using the 3-0 monofilament continuous suture. The continence of the pouch was tested by pinching the catheters and instilling 250–300 ml of saline solution. Finally, in the right pelvic region, the previously catheterized and tapered ileum is exteriorized and fixed to the skin using simple vycril 3-0 sutures (Fig. 9, Fig. 10, Fig. 11).

The ureteral stents and the cystostomy drain were removed 15 days after surgery, after confirming the patient´s ability and comprehension of self-catheterization. The patient was then discharged.

## 4 Follow-up

The patient attended her first follow-up visit 3 months after surgery. The patient was asymptomatic apart from a urine infection and a white lesion in the introitus. A PET-CT scan was performed, revealing a suspicious looking lesion in the right pelvis, affecting the vaginal introitus, with a SUV max of 6. A vulvar biopsy was performed, as well as a transrectal ultrasound guided biopsy with a Trucut needle of the right perianal wall lesion. The result of both biopsies gave a well-differentiated infiltrating squamous carcinoma. On June 2016, the patient underwent a radical vulvectomy, anal amputation and pelvic lesion resection with immediate vulvar reconstruction using the gracilis flaps as a salvage surgery. After an 11-day hospital stay, the patient was discharged on July 6th 2016. One month later, the patient was completely asymptomatic and the imaging studies were negative. Three months later, the patient attended her second follow-up visit. The CT scan and SCC markers were negative, and there were no signs of graft rejection. The patient tolerated her permanent colostomy and intermittent self-catheterization, and presented a normal renal function. The next follow-up visits took place 6 months later and later onannually.

## 5 Discussion

To day, the patient has come to our consultation for two annual follow-up visits. The CT scans, SCC tumor marker, and renal function panel were all within the normal range. Apart from the various episodes of uncomplicated urinary infections the patient experienced the first few months following the IP construction surgery. Complications were treated using oral antibiotherapy, no other problems were observed. The patient isused to the intermittent self-catheterization (every 4 h) and refers a successful daytime continence and an overall good quality of life.

The ileal conduit, as described by Bricker, was once the gold standard urinary diversion for the gynecologic oncology patient with a history of pelvic irradiation, as it presented minimal electrolyte disturbances and it was easily constructed. However, it has long been criticized due to the lack of urinary continence and the appearance of renal dysfunction [12]. Therefore, in order to maintain a tolerable quality of life for the patient, a CCUD would be a better choice. In addition to the IP being a continent reservoir; its construction uses an ascending colon segment that is located outside the radiation field, in contrast to the Kock pouch, decreasing the rate of long-term stenosis due to the ureter being tunneled into a non-irradiated bowel segment [13].

On the other hand, other surgeons suggested constructing a urinary reservoir using the transverse colon in patients with a history of pelvic radiation in order to avoid the use of irradiated bowel segments and damaged ureters. Contradicting what was previously mentioned, during pelvic radiotherapy for cervical cancer, the cecal pole, parts of the ascending colon, appendix and ileum are exposed to considerable doses of radiation. However, using only the transverse colon will not provide continence, which makes it a less popular alternative. Mannel et al. published in 1990 a series of 10 patients with previous pelvic irradiation who received an IP reservoir. Initially, there was no report of postoperative complications and all patients were reported to be continent. However, five years later, Mannel et al. reported on 37 irradiated female patients with a similar history, presenting ureteral stricture in 3% of the cases, and hydronephrosis in 7% [14]. Both studies had an average follow-up of 11 months, but a higher complication rate was observed in the larger study. There are currently no published studies with a follow-up of more than 10 years in irradiated patients with CCUD; nevertheless up to 25% of the patients had to be re-intervened during the limited follow-up [15]. Radiation damage is known to increase with time; this is why a longer follow-up time is required in order to evaluate the surgical complication rate.

Al Awamlh et al. reports that 89% of all patients that have undergone a CCUD were still continent after an average follow-up of 3 years. Despite the IP successful continence as a urinary reservoir, the CCUD remains infrequently performed and long-term observation has not been described [16]. This also supports the fact that there is a lack of long-term follow-up.

One final aspect of the IP which is rarely reminded is the possibility of developing an adenocarcinoma in the urinary diversion. It is a known complication, and is based on the pathogenesis theory of nitrosamine carcinogen exposure to urine. It occurs in approximately 10% of the patients following ureterosigmoidostomy, however Bell et al. describe a case of adenocarcinoma in Indiana pouch constructed 8 years prior to the diagnosis [17]. There is a suggested surveillance protocol using cystoscopy screening starting 10 years after the creation of the urinary diversion [18], which we will also consider adopting once we reach the 5 year follow-up instead of waiting for 10 years, having evidence that there are some occurring cases before that time span.

## 6 Conclusion

There can be nothing conclusive about a case report regarding a single patient; however, a review of the already published literature has revealed the need for long-term studies in order to observe the possible complications that may appear years after.

## Sources of funding

This work received financial support from Medtronic University Chair for Training and Surgical Research. (University Jaume I (UJI), Castellon, Spain).

## Ethical approval

Approval by the Ethics and research committee of our institution (CEIm) was obtained. Ref. 012/2018.

## Consent

Written informed consent was obtained from the patient for publication of this case report and accompanying images. A copy of the written consent is available for review by the Editor-in-Chief of this journal on request

## Author contribution

Study concepts: Antoni Llueca.

Study design: Antoni Llueca.

Data acquisition: Anna Serra, Paula Ponce.

Quality control of data and algorithms: Yasmin Maazouzi.

Data analysis and interpretation: Miguel Rodrigo.

Manuscript preparation: Antoni Llueca.

Manuscript editing: Antoni Llueca, Yasmin Maazouzi.

Manuscript review: Carmen Garau.

## Registration of research studies

This is not a first-in-man case report and thus cannot be registered.

## Guarantor

Dr. Llueca is the guarantor of the paper.

## Provenance and peer review

Not commissioned, externally peer-reviewed

## Declaration of Competing Interest

The authors declare no conflict of interest.
